# Factors Associated with Suicidal Behavior in Adolescents: An Umbrella Review Using the Socio-Ecological Model

**DOI:** 10.1007/s10597-024-01368-2

**Published:** 2024-11-02

**Authors:** Virginia Prades-Caballero, José-Javier Navarro-Pérez, Ángela Carbonell

**Affiliations:** Department of Social Work and Social Services, Universitat de València, Valencia, Spain

**Keywords:** Adolescence, Risk factors, Protective factors, Suicide, socio-ecological model, Umbrella review

## Abstract

Adolescent suicide is a critical social issue with profound and lasting individual and collective consequences. This umbrella review examines factors associated with adolescent suicidal behavior through the socioecological framework of prevention and seeks to identify gaps in the existing literature. The review followed the Preferred Reporting Items for Systematic Reviews and Meta-Analyses (PRISMA) guidelines and included a comprehensive search of the of the Web of Science, Scopus, and Cochrane databases in both English and Spanish, covering the period from 2018 to 2024, using terms related to adolescence and suicidal behavior. Out of 6,138 articles identified, 37 met the quality criteria and were selected for analysis. The studies highlighted individual risk factors such as age, gender, belonging to ethnic or gender minorities, emotional disorders, self-destructive behavior, emotional regulation, and experience of physical or emotional pain. Interpersonal factors such as parental relationships, sexual abuse, social isolation, peer pressure, and loneliness were also found. However, the review revealed a worrying lack of studies on societal and community factors and a paucity of research focusing on protective factors. The study highlights the need to include factors related to the physical and social environments that influence health and behavior in future research, as well as to enhance the resources and strengths of adolescents.

## Introduction

Suicidal behavior has been recognized as a social and public health problem of growing concern and has gained interest as an object of study in various scientific disciplines (Lodha et al., 2023; Palanbek-Yavaş & Baysan, 2024). According to the World Health Organization (WHO, 2024), approximately 702,000 suicides are reported globally each year, which is equivalent to one death by suicide every 40 s. Currently, suicide is the third leading cause of death among adolescents and young people aged 15 to 29 years. This implies that almost one-third of all suicide deaths occur in adolescents and young people, with the incidence steadily increasing among the youngest (WHO, 2021). Although there is no universally accepted figure for adolescents, WHO (2024) estimates that, globally, there are approximately 20 suicide attempts for every completed suicide. This reality underscores the urgency of developing and implementing evidence-based strategies for prevention and intervention in suicidal behavior during this critical developmental stage.

Despite decades of research, the increasing rates of adolescent suicide demonstrate that the ability to predict and prevent this phenomenon has not significantly improved (Standley, 2022). Recently, the scientific literature has questioned the efficacy of commonly applied preventive efforts in schools, social services, and primary health care (Itzhaky et al., 2022; Wasserman et al., 2021). While important advances have been made, limitations in their implementation and evaluation still persist. Studies (Cha et al., 2018; Seag et al., 2022) have identified that one of the fundamental problems is the gap in universal screening and the limitations of a holistic identification of risk factor heterogeneity. In this regard, defining the multifactorial etiology of suicidal behavior remains a confounding task with many open questions (Berman & Silverman, 2017).

For Pelkonen et al. (2011), research on risk factors for adolescent suicide provides the basis for suicide prevention. Despite the prolific existing evidence on conditions that increase vulnerability, the predominant approaches in research and prevention have proven insufficient to address the complexity of risk (Standley, 2022). Studies often focus on explanations that offer an individualistic, objective, and pathological view of suicide, neglecting the influence of social contexts and ecological factors (Hughes et al., 2023). In addition, protective factors have often been omitted from the literature or analyzed only in specific subpopulations (Nielassoff et al., 2023). According to Marraccini et al. (2021) and Wasserman et al. (2021), understanding the interaction and combination of all associated risk and protective factors is essential for developing and implementing effective preventive strategies, identifying the nature of suicidal ideation, and establishing a comprehensive approach to adolescent suicide.

From a critical suicidology approach, previous research (Kirmayer, 2022; White, 2017) has advocated for multilevel approaches to the prevention, diagnosis, and intervention of suicidal behavior. The Social-Ecological Suicide Prevention Model (SESPM) (Cramer & Kapusta, 2017) has been proposed as the most comprehensive framework for addressing this phenomenon. Inspired by Bronfenbrenner’s (1979, 2013) ecological model, this perspective views suicide as a multidimensional and multicausal phenomenon, influenced by a variety of risk and protective factors that interact through different systems. These systems are shaped by contemporary theories of suicide risk, incorporating perspectives at the individual (e.g., Shneidman, 1981), interpersonal or relational (e.g., Joiner, 2005; Van Orden et al., 2010), community (e.g., Castro & Kintzle, 2014), and societal or environmental (e.g., Durkheim, 1897) levels. Consequently, the model encompasses a range of factors, including family conflict or violence, biological and sociodemographic elements, psychiatric diagnoses, feelings of hopelessness, social stigma, place of residence, poverty, and barriers to healthcare access. Additionally, protective factors such as the presence, use, and perception of positive social support, school support, and intervention programs are identified as crucial. The realities and contexts in which adolescents live and interact offer opportunities to identify and understand associated protective factors, providing valuable insights for prevention and intervention (Standley & Foster-Fishman, 2021). In this context, authors such as Mueller et al. (2021) and Wiglesworth et al. (2022) argue for the investigation of cultural assets, subjective experiences of adolescents, and the intersectionality of factors.

Although the SESPM provides a comprehensive framework in the field of prevention, it is considered a preliminary and adaptive model, suggesting that it may require adjustments to account for the specific characteristics of each population or context (Cramer & Kapusta, 2017). Nevertheless, the SESPM offers an organizational structure that can guide the development of future systematic reviews on risk and protective factors, thereby facilitating the creation of appropriate prevention programs for at-risk groups, such as adolescents. Along these lines, Abrutyn and Mueller (2021) have emphasized the importance of adopting interdisciplinary and transdisciplinary approaches to suicide intervention and research. This is particularly relevant during adolescence, as the early identification of risk factors, along with the implementation of comprehensive and coordinated interventions, can significantly influence the developmental trajectory, mental health outcomes, and social well-being of adolescents (Marzec et al., 2021). However, most studies and interventions have been developed from disciplines such as psychology, psychiatry, epidemiology, and sociology. As a result, fragmented prevention strategies have often failed to adequately address risk factors at the community and societal levels (Standley, 2022).

All in all, suicide prevention in adolescence continues to face significant challenges. Following the policy cycle framework, accurately identifying the nature and scope of the problem is the first step in designing appropriate interventions (Chindarkar et al., 2022; Poblador & Lagunero-Tagare, 2023). This underscores the need to further identify risk factors and protective elements against suicidal behavior at this stage of development (Fonseca-Pedrero et al., 2022). Although several systematic reviews analyze factors associated with suicidal behavior, the diversity of settings, populations, and research approaches complicates obtaining a comprehensive picture of the topic and utilizing the large amount of available data to design and implement effective prevention policies. Therefore, this study conducts a systematic umbrella review with the aim of analyzing factors associated with suicidal behavior in adolescents from the socioecological framework of suicide prevention and identifying existing gaps in the literature. This review seeks to contribute to a holistic understanding of suicidal behavior by providing a rigorous, interdisciplinary, and critical analysis that serves as a foundation for future research and comprehensive intervention strategies, encompassing all relevant factors and disciplines involved, and allowing for a paradigm shift in the study of suicide.

## Materials and Methods

An umbrella review, or systematic review of systematic reviews, is conducted by adhering to PRISMA (Preferred Reporting Items for Systematic Reviews and Meta-Analyses) standards (Page et al., 2021). The main objectives of this type of review are to address the growing number of systematic reviews and meta-analyses, to provide a clear and comprehensive evidence base regarding its findings, as well as gaps and research needs (Choi & Kang, 2023).

### Search Strategy

The ‘Population, Exposure, Outcome’ (PEO) framework, as proposed by Moola et al. (2015), was used to develop the search strategy, facilitate the literature search, and establish the inclusion criteria. In this framework, the Population refers to adolescents, Exposure denotes suicidal behavior, and Outcome encompasses risk and protective factors.

The studies included in this review are systematic reviews, with or without meta-analysis, focusing on the adolescent population (ages 12–20) as the dependent variable and factors associated with suicide as the independent variable. All the studies had to meet the following criteria: (1) published between 2018 and 2024 to ensure recency and relevance; (2) published in peer-reviewed journals; (3) available in English or Spanish, the most common languages in suicide research and among the largest in terms of number of speakers worldwide; and (4) conducted in any geographic setting. We excluded studies that: (1) did not directly analyze factors associated with suicide; (2) focused solely on youth or mixed samples without segmenting the results by developmental stage; (3) focused exclusively on parasuicidal behaviors; (4) analyzed the moderation of the relationship between factors and adolescent suicide; and (5) were classified as gray literature.

The literature search was conducted on February 2, 2024, through the Web of Science, Scopus, and Cochrane databases. The primary search terms encompassed concepts related to suicidal behavior, adolescence, and associated factors. Boolean operators (OR and AND) were employed, along with truncations, using key descriptors in the title, abstract, and keyword fields. Table 1 presents the search strategy used.

**Table 1:** Search strategy

Language	Search string
English	(adolescen* OR teen*) AND suicid* AND (“associated factor*” OR “protective factor*” OR “risk factor*”)
Spanish	“adolescen* AND suicid* AND (“factor* asociado*” OR “factor* protector*” OR “factor* de protección*” OR “factor* de riesgo*”)

### Data Extraction and Analysis

To analyze and synthesize the obtained data, the identified documents were selected and categorized using the PRISMA flow diagram (Page et al., 2021). Each selected article was examined based on the following descriptive aspects: authors and year of publication, study design, follow-up, databases, included articles, population, and type of factors analyzed according to the SESPM (Cramer & Kapusta, 2017). Subsequently, a narrative and interpretative synthesis of the results was conducted through a deductive-mixed content analysis (Finfgeld-Connett, 2014). In analyzing the text data, the qualitative data analysis software MAXQDA was used.

### Quality Assessment

To ensure quality and transparency in the review process, the PRISMA guidelines were adhered to (Page et al., 2021). The validity of umbrella reviews depends on the coverage and quality of the available systematic reviews and meta-analyses, as well as the primary studies included (Belbasis et al., 2022). Thus, we assessed the risk of bias and the methodological quality of the set of included articles to determine the validity of the results. We used the Critical Appraisal Skills Program (CASP) (2023) - Systematic Review Checklist tool (Long et al., 2020; Singh, 2013), which consists of 10 questions to critically evaluate systematic review reports. Scores were categorized into three levels: low quality (0–3 points), moderate quality (4–7 points), and high quality (8–10 points). No papers were excluded at this stage.

The online tool Parsifal (Freitas, 2014), developed in the context of software engineering to help researchers conduct systematic reviews, was used to classify, sort, and assess the quality of the articles. The literature search, study selection, data extraction, content analysis, and quality assessment were performed by two study authors (VPC, AC) independently. In the event of disagreement during the process, consensus was reached through discussion with a third author (JJNP), which ensured objectivity and reduced review bias (Wainwright & Macnaughton, 2013).

## Results

### Number of Studies Included

**Fig. 1:**
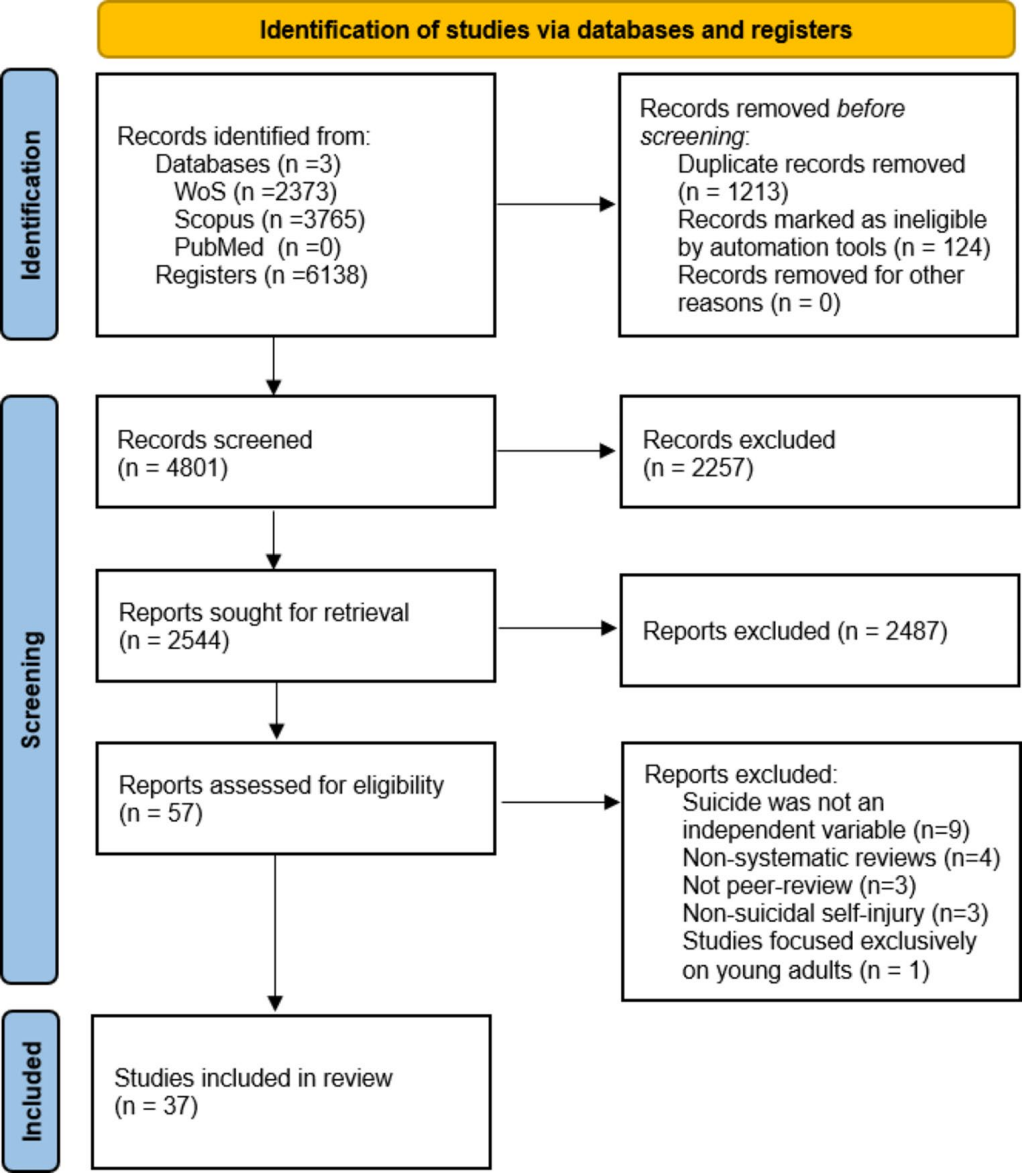
PRISMA 2020 Flow Diagram. *Note* Adapted from Page et al. (2021)

The results of the search and selection process are presented in Fig. 1 below. In total, 37 articles were included in the review.

### Characteristics of Included Studies

Table 2 lists the main methodological and quality characteristics of the included studies. The 37 articles analyzed and included in this umbrella review were rated as having high or moderate quality. Thirteen articles (35.13%) focus exclusively on the adolescent population, while 24 articles (64.86%) also include young individuals up to 20 years of age.

**Table 2:** Characteristics of studies included in the umbrella review

Authors (year)	Study design*	Years	Databases	Included articles	Population	Associated factors		MQ**
						Risk factors	Protective factors	
Angelakis et al. (2020)	SRMa	1980–2019	MedLine, PsychInfo, Embase, Web of Science and CINAHL	79	Adolescents and young adults	Interpersonal		High
Ati et al. (2020)	SR	2015–2019	ScienceDirect EBSCO, Direct Open Acces Journal and ProQuest	66	Adolescents	Individual, Interpersonal	Individual, Interpersonal	High
Benarous et al. (2018)	SR	1990–2017	PubMed, MedLine, PsychINFO, PsychINDEXPlus and Dissertation Abstracts	27	Adolescents	Individual	Individual	High
Buelga et al. (2022)	SR	2018–2022	Web of Science, Scopus, PsycInfo, PsycArticles and PubMed	21	Adolescents	Interpersonal		High
Calati et al. (2019)	SR	To 2018	PubMed	40	General population divided by sector	Interpersonal		High
Calderaro et al. (2022)	SRMa	To 2020	PubMed, PsycINFO and EMBASE	20	Adolescents	Interpersonal		High
Castellví et al. (2020)	SRMa	To 2017	Cochrane, Library, PubMed/MedLine, PsychINFO, EMBASE, Web of Science and OpenGrey	14	Adolescents and young adults	Individual, Community		High
Chen et al. (2022)	SRMa	1900–2020	PubMed, PsycINFO, MedLine and EMBASE	59	Adolescents and young adults	Interpersonal		High
Chiu et al. (2018)	SRMa	To 2017	EMBASE, PubMed, PsycINFO, ProQuest Dissertations & Theses A&I, Wanfang Data and China Knowledge Resource Integrated Database	10	Adolescents	Individual		High
Cuesta et al. (2021)	SR	2010–2020	CINAHL, Scopus, PubMed, Google Scholar and Cuiden	18	Adolescents	Individual, Interpersonal		High
Del Carpio et al. (2021)	SR	To 2020	MedLine, PsycINFO, Web of Science and EMBASE	21	Adolescents	Interpersonal		High
Domínguez-García & Fernández-Berrocal (2018)	SR	To 2018	Web of Science, Scopus, MedLine, PubMed, PsychINFO, ProQuest, Riuma, Dialnet and Google Schoolar	25	General population divided by sector	Interpersonal	Individual	High
Gelvez-Gafaro et al. (2022)	SR	2012–2022	EBSCO, Redalyc, PubMed, ScienceDirect, ProQuest and Scielo	80	Adolescents and young adults	Individual, Interpersonal		High
Goodday et al. (2019)	SR	To 2017	MedLine, CINAHL, EMBASE, PsycINFO, Web of Science and grey literature	54	Adolescents and young adults	Interpersonal		High
González-Sancho & Picado (2020)	SR	1995–2017	Redalyc, Scielo, Repositorio Nacional Kímuk del Consejo Nacional de Rectores and Journal Storage	78	Adolescents and young adults	Individual, Interpersonal, Community	Individual, Interpersonal	High
Hatchel et al. (2019)	SRMa	1990–2017	PsycINFO, EBSCO and PubMed	44	Adolescents and young adults	Individual		High
Hernández-Bello et al. (2020)	SR	2009–2017	Cuiden, Lilacs, PubMed, MedLine, Scielo, Science Direct, Scopus and Ebsco	23	Adolescents	Individual, Interpersonal	Individual, Interpersonal	High
Hinze et al. (2019)	SR	1961–2018	EMBASE, PsychINFO, MedLine, CINAHL, PubMed, Web of Science and Scopus	25	Adolescents	Individual		High
Howarth et al. (2020)	SRMa	To 2019	MedLine, PsychINFO, EMBASE, CINAHL and Cochrane.	15	Adolescents and young adults	Interpersonal		High
Jha et al. (2023)	SRMa	2008–2022	PsycINFO, IEEE Xplore, ACM Digital Library and PubMed	25	Adolescents and young adults	Individual, Interpersonal		High
Karanikola et al. (2019)	SR	2007–2018	MedLine, CINAHL, PsychINFO, Scopus and Cochrane.	20	Adolescents and young adults	Individual		High
Kearns et al. (2020)	SR	2017	PsychINFO, PubMed and Web of Science	10	Adolescents and young adults	Individual		Moderate
Kohlbeck et al. (2020)	SRMa	2012–2016	WVDRS	20	Adolescents	Individual		High
Leigh et al. (2022)	SRMa	To 2022	Embase, PsychINFO y MedLine	16	Adolescents and young adults	Individual		High
Liu et al. (2019)	SRMa	To 2018	EMBASE, PubMed, ProQuest and China Knowledge Resource Integrated Database.	12	Adolescents	Individual		High
Londoño and Cañón (2020)	SR	2014–2019	PubMed, ProQuest, Scopus and Dialnet	54	Adolescents	Individual		High
Marconi et al. (2023)	SR	To 2022	PubMed, Scopus, PsycINFO and Web of Science	21	Adolescents and young adults	Individual		High
Miranda-Mendiazábal et al. (2019)	SRMa	1995–2017	Cochrane Library, Medline, PsychINFO, EMBASE and Web of Science	77	Adolescents and young adults	Individual		High
Nesi et al. (2021)	SRMa	To 2020	PsycINFO, MedLine and CINAHL	61	Adolescents and young adults	Individual, Interpersonal		High
Quarshie et al. (2020)	SR	1950–2019	MedLine, PsycINFO, PubMed, African Journals OnLine and African Index Medicus	74	Adolescents and young adults	Individual, Interpersonal		High
Russell et al. (2021)	SR	To 2020	MedLine, Scopus, PsycINFO, Web of Science, The Social Science Index and ProQuest	5	Adolescents and young adults	Interpersonal		High
Shahram et al. (2020)	SR	2008–2018.	Cinahl, MedLine, PsycArticles, PsycINFO, PsycExtra, Social Work Abstracts and Google Schoolar.	37	Adolescents and young adults		Individual, Interpersonal	High
Silva et al. (2024)	SRMa	To 2022	PubMed, Biblioteca Virtual em Saúde (BVS), Scielo, LILACS, PEPSIC, PsycInfo and Scholar Google	6	Adolescents	Individual		High
Steare et al. (2023)	SR	1991–2022	MedLine, PsycINFO, ERIC and Web of Science	52	Adolescents	Community		High
Soto-Sanz et al. (2019)	SRMa	To 2017	Cochrane, Embase, MedLine, PsychINFO and Web of Science	65	Adolescents and young adults	Individual		High
Wang et al. (2023)	SR	To 2020	PubMed, Web of Science, PsycINFO and Cochrane	12	Adolescents and young adults	Individual		High
Wilson et al. (2022)	SR	To 2021	MedLine, Embase, PsycINFO, CINAHL and Scopus	35	Adolescents and young adults	Interpersonal		High

*Note* *SR = Systematic Review. SRMa = Systematic Review with Meta-Analysis. **MQ = Methodological Quality

### Associated Factors with Suicidal Behavior in Adolescents

Following the SESPM classification, there are four descriptive categories of factors associated with suicidal behavior in adolescents - individual, interpersonal, community and societal factors- which are organized according to whether they serve as risk factors or protective factors.

#### Risk Factors

Of all the articles analyzed, 36 (97.29%) focus on reviewing the risk factors for suicidal behavior in adolescents. These factors refer to identifiable characteristics or circumstances within individuals, groups, or communities, as well as within their social, political, historical, and cultural contexts, which are associated with a greater probability of the phenomenon occurring.

##### Individual Risk Factors

Twenty-seven of the studies included in this systematic review (72.97%) address individual risk factors. These factors refer to individual characteristics or circumstances that increase vulnerability to suicidal behaviors (Ati et al., 2020; Benarous et al., 2018; Castellví et al., 2020; Chiu et al., 2018; Cuesta et al., 2021; Domínguez-García & Fernández-Berrocal, 2018; Gelvez-Gafaro et al., 2022; González-Sancho & Picado, 2020; Hatchel et al., 2019; Hernández-Bello et al., 2020; Hinze et al., 2019; Jha et al., 2023; Karanikola et al., 2019; Kearns et al., 2020; Kohlbeck et al., 2020; Leigh et al., 2022; Liu et al., 2019; Londoño & Cañón, 2020; Marconi et al., 2023; Miranda-Mendiazábal et al., 2019; Nesi et al., 2021; Silva et al., 2024; Soto-Sanz et al., 2019; Wang et al., 2023). As noted by Cuesta et al. (2021) and Hernández-Bello et al. (2020), these factors may vary in intensity from one individual to another and include aspects such as age, gender, sexual orientation, gender identity, ethnicity, or religion. Additionally, they encompass emotion management skills, feelings of hopelessness, social isolation, affective or mood disorders, and other physical or psychological ailments.

###### Sociodemographic Risk Factors

As sociodemographic factors, age and gender have been reviewed by five of the included studies (Cuesta et al., 2021; Hernández-Bello et al., 2020; Kohlbeck et al., 2020; Londoño & Cañón, 2020; Miranda-Mendiazábal et al., 2019). Regarding age, the study by Kohlbeck et al. (2020) reveals that suicide risk also varies by age group. This research analyzes characteristics shared among all adolescents (10–17 years) and compares particularities between subgroups: pre-secondary (10–13 years) and secondary (14–17 years). It is observed that adolescents aged 14 to 16 years face greater risk due to personal and social crises specific to this stage. According to Cuesta et al. (2021) and Londoño and Cañón (2020), this is related to the “adolescent crisis,” which involves hormonal changes, the search for individuality and independence, the establishment of healthy relationships with peers, and non-normative crises such as parental divorce or changes in body, thoughts, and feelings.

Studies by Miranda-Mendiazábal et al. (2019), Hernández-Bello et al. (2020), and Londoño and Cañón (2020) agree that girls have a significantly higher risk of suicide attempts compared to boys. However, it is noted that boys have a risk of dying by suicide that is nearly three times higher than that of girls, possibly due to greater impulsivity and the use of more lethal methods (Miranda-Mendiazábal et al., 2019). The study by Quarshie et al. (2020) reports that, in Africa, suicide among women is interpreted as a protest against socially sanctioned abuse and oppressive control, whereas self-harm and suicide in men represent a search for lost masculinity. Gender-based dating violence and experiencing interpersonal difficulties are also associated with higher rates of suicide attempts among women (Miranda-Mendiazábal et al., 2019). Kohlbeck et al. (2020), in a study with adolescent females, note that sexual abuse, as part of childhood trauma, contributes to suicidal behavior among females. In addition, disorders that increase the risk of suicide attempts exclusively among females include bipolar disorder and eating disorders. In contrast, the emotional factors significantly associated with suicide attempts among males are disturbances and hopelessness. In addition to these differences, risk factors common to both genders include having experienced suicidal ideation at some point, previous suicide attempts and/or a history of emotional disorders such as anxiety, major depressive disorder, or personality disorders, as well as alcohol and other drug abuse (Miranda-Mendiazábal et al., 2019).

Seven studies (Castellví et al., 2020; Cuesta et al., 2021; González-Sancho & Picado, 2020; Hatchel et al., 2019; Marconi et al., 2023; Soto-Sanz et al., 2019; Wang et al., 2023) examine the association between sexual and gender minority membership and suicidal behavior in adolescents. These studies agree that factors such as the social construction of gender and conflicts related to sexuality increase the risk of suicidal ideation or attempts in adolescents. Lesbian, gay, bisexual, transgender, and questioning (LGBTQ) adolescents have a significantly higher risk of suicidal thoughts or behaviors compared to their non-LGBTQ peers. This is primarily due to exposure to external stigma, such as peer victimization, and other variables such as identity, personality traits, community, and family and social acceptance, which are linked to psychological well-being (Hatchel et al., 2019).

Only one study (Cuesta et al., 2021) examines minority ethnicity as a individual risk factor. This risk can be explained by the stigma, discrimination, and victimization experienced from the rest of society, according to minority stress theory (Hatchel et al., 2019; Wang et al., 2023), rather than being attributable to identity or internal conflicts. According to Hatchel et al. (2019), the association between suicidal behavior and adolescents belonging to sexual and gender minorities is stronger in countries or territories where there is a negative social perception towards these individuals or where the laws and norms concerning this group are more restrictive. The sociohistorical construction of the meaning of being male or female leads to discriminatory acts of rejection and violence against these individuals, increasing their suicide risk (González-Sancho & Picado, 2020). According to González-Sancho and Picado (2020), these factors serve as warning signs, and the seriousness of the situation is exacerbated when these variables occur simultaneously and in combination within the environments faced by adolescents, thereby confirming the multifactorial complexity of the phenomenon.

Studies analyzing the association between asocial behaviors and suicidal behavior in adolescents emphasize the relationship with school failure and dropout, as well as legal problems. Castellví et al. (2020) and Cuesta et al. (2021) report a positive correlation between school failure and suicidal behavior in adolescents, although they do not address the different dimensions of school failure, such as low academic performance, school dropout, expulsion, and grade repetition. Cuesta et al. (2021) indicate that the risk of suicidal behavior increases significantly when low academic performance is combined with school dropout, which can lead to feelings of personal and familial failure, increasing the risk of suicide by more than six times. Additionally, Soto-Sanz et al. (2019) demonstrate a positive association between legal problems, such as contact with the police or the commission of serious crimes, and suicidal behavior in adolescents. However, the review cannot determine how these dimensions interact with each other or with other risk factors, highlighting the need to explore the underlying mechanisms that explain this relationship.

###### Psychiatric Risk Factors

Despite the fact that all the studies reviewed have shown that emotional disorders are not consistently reliable predictors of suicidal behavior in adolescents, a large percentage of research continues to focus on this association. Specifically, 10 of the included studies review affective or mood disorders as individual risk factors (Benarous et al., 2018; Cuesta et al., 2021; Domínguez-García & Fernández-Berrocal, 2018; Gelvez-Gafaro et al., 2022; González-Sancho & Picado, 2020; Hernández-Bello et al., 2020; Jha et al., 2023; Leigh et al., 2022; Londoño & Cañón, 2020; Soto-Sanz et al., 2019). Most of these studies report a significant association between internalizing symptoms and future suicide attempts or completed suicide in adolescents. Soto-Sanz et al. (2019), Jha et al. (2023), Londoño and Cañón (2020), and Leigh et al. (2022) find that the emotional problems most commonly associated with suicidal behavior in adolescents are depression, anxiety, and social anxiety.

Eight articles examine the relationship between risk behaviors, such as alcohol and other psychoactive substance use, and suicidal behavior in adolescents (Ati et al., 2020; Cuesta et al., 2021; Gelvez-Gafaro et al., 2022; Hernández-Bello et al., 2020; Jha et al., 2023; Karanikola et al., 2019; Londoño & Cañón, 2020; Soto-Sanz et al., 2019). The growing interest in this area is due to the decreasing age of onset of substance use and its association with suicidal behavior (Cuesta et al., 2021; Hernández-Bello et al., 2020; Jha et al., 2023; Londoño & Cañón, 2020). Gelvez-Gafaro et al. (2022) report that the risk of addictions increases due to the normalization of consumption, the search for autonomy, the need to belong, and the pursuit of pleasure. In addition, impaired impulse control, social interaction, and behavior associated with substance use elevate suicidal risk. The review by Karanikola et al. (2019) finds that adolescents who use cannabis are 6–16 times more likely to attempt suicide than non-users. Londoño and Cañón (2020) report that this risk increases up to 4.1 times when use begins before age 13, whereas, in adolescent users of other substances, suicide attempts are up to twice as common.

The study by Nesi et al. (2021) investigates the relationship between the excessive use of social networks and suicidal behavior in adolescents. Excessive use of these platforms, which manifests as a disproportionate dedication of time and energy, can lead to addiction-like symptoms and cause significant impairment in adolescents. Online bullying and sexting represent additional stressors, as adolescents may be publicly victimized at any time and by anonymous individuals. Female adolescents, who tend to spend more time on and be more active on social networks, are at a higher risk of suicidal behavior.

Reviews (Ati et al., 2020; Cuesta et al., 2021; Hernández-Bello et al., 2020; Soto-Sanz et al., 2019; Karanikola et al., 2019) reveal that addictive behaviors can cause adolescents to perceive certain situations as more stressful than usual. This often results in an inability to apply effective coping styles and the development of maladaptive behaviors, which may lead to considering suicidal behavior as a way out. Despite these troubling findings, the study by Londoño and Cañón (2020) underscores the need to further investigate the relationship between addictive behaviors and suicidal behavior, taking into account variables such as cultural norms and impulsivity from a gender perspective. Furthermore, it highlights that this risk may be higher in vulnerable groups, such as adolescents in conflict with the law, members of the LGBTQ community, and hospitalized adolescents. Therefore, further studies are required to address these issues in a comprehensive manner (Karanikola et al., 2019).

###### Psychological Risk Factors

Regarding psychological risk factors, seven articles (Benarous et al., 2018; Cuesta et al., 2021; Domínguez-García & Fernández-Berrocal, 2018; Hernández-Bello et al., 2020; Leigh et al., 2022; Londoño & Cañón, 2020; Silva et al., 2024) examine the relationship between emotion regulation and suicidal behavior in adolescents. Psychological factors associated with suicide risk include low self-esteem, hopelessness, fears about the future, stress, self-perception problems, lack of optimism, and difficulties coping with challenging situations (Domínguez-García & Fernández-Berrocal, 2018; Hernández-Bello et al., 2020; Londoño & Cañón, 2020; Silva et al., 2024). Ineffective coping, especially emotion-focused and avoidant coping, is positively associated with suicidal behavior, whereas problem-focused coping is negatively related to suicidal behavior (Ati et al., 2020). Cuesta et al. (2021) and Ati et al. (2020) highlight that negative self-perception, particularly in relation to weight and eating disorders, significantly affects suicidal behavior, with a greater impact on women.

Four studies analyze sleep and unhealthy lifestyle habits as risk factors for suicidal behavior in adolescents (Ati et al., 2020; Chiu et al., 2018; Kearns et al., 2020; Liu et al., 2019). During adolescence, changes in sleep quantity and quality are significant. Adolescents tend to go to bed and wake up late, which often conflicts with school schedules (Kearns et al., 2020; Liu et al., 2019). This pattern is attributed to several factors, including prolonged use of cell phones and exposure to artificial light before bedtime, which hinder the transition to sleep. Dependence on social networks such as TikTok also contributes to adolescents spending more time online, delaying bedtime. As a result, teens experience poor sleep patterns, which negatively affect their overall health and well-being. Lack of sleep reduces serotonin levels, impairs impulse control and judgment, and decreases problem-solving skills. Chiu et al. (2018) demonstrate that increasing sleep duration by one hour can reduce the risk of suicidal ideation by up to 11%, suggesting that improving sleep habits could be an effective intervention to prevent suicide in adolescents.

Leisure time in adolescents is frequently associated with suicide attempts, especially when leisure time is largely sedentary. Studies, such as that of Ati et al. (2020), emphasize that this correlation underlines the need to effectively manage leisure time and promote attractive and stimulating activities. Regional and local institutions have the opportunity to invest in programs and spaces dedicated to leisure that offer healthy and exciting alternatives, such as physical activities, the creation and maintenance of healthy interpersonal relationships, and creative and cultural activities. By encouraging active and socially enriching leisure time, adolescents’ mental and physical health can be improved, their emotional states can be balanced, and the risk of suicide attempts associated with sedentary leisure and social isolation can be reduced.

Only Hinze et al. (2019) explore the relationship between pain and suicidal behavior in adolescents. Their results indicate that while acute pain, whether physical or psychological, may motivate help-seeking and promote recovery, chronic pain is associated with greater distress and self-destructive behaviors. This is because acute pain is usually temporary and related to a specific injury or event, whereas chronic pain is persistent and can generate long-term hopelessness. Resilience to pain is strengthened when it is perceived as temporary and treatable, but chronic pain significantly affects adolescents’ daily lives and academic, social, and leisure activities, thus elevating suicidal risk.

##### Interpersonal Risk Factors

A total of 17 studies (45.95%) review interpersonal risk factors, defined as conditions and circumstances related to the adolescent’s relational environment that increase his or her vulnerability to suicide. The included studies show that suicide risk is related to problems associated with the family environment, including violence, conflicts, family history of mental illness and suicide, as well as relationship instability (Ati et al., 2020; Gelvez-Gafaro et al., 2022; Goodday et al., 2019; González-Sancho & Picado, 2020; Hernández-Bello et al., 2020; Jha et al., 2023; Londoño & Cañón, 2020). A connection with the adolescent’s relationships with his or her peer group, such as sentimental breakups, is also observed (Ati et al., 2020; Buelga et al., 2022; Cuesta et al., 2021; Gelvez-Gafaro et al., 2022; González-Sancho & Picado, 2020; Londoño & Cañón, 2020; Wilson et al., 2022) or social isolation or withdrawal (Ati et al., 2019; Calati et al., 2019; Domínguez-García & Fernández-Berrocal, 2018; González-Sancho & Picado, 2020).

Eight of the ten included studies review the impact that the family has on suicidal behavior in adolescents (Ati et al., 2020; Cuesta et al., 2021; Chen et al., 2022; Gelvez-Gafaro et al., 2022; Goodday et al., 2019; González-Sancho & Picado, 2020; Hernández-Bello et al., 2020; Jha et al., 2023; Londoño & Cañón, 2020) and find that it is the most significant risk factor for adolescent suicide. The family, as the first socialization environment, is fundamental in the development of communication processes, the construction of trusting relationships and the internalization of values such as respect and solidarity. When events arise that disorganize or fracture this family structure, adolescents may manifest disruptive behaviors, including suicidal behaviors (Goodday et al., 2019). Factors such as low parental schooling, drastic changes in living standards, family disorganization, single parenthood (Cuesta et al., 2021), physical or emotional neglect, unemployment, and intrafamily conflict (Ati et al., 2020; Gelvez-Gafaro et al., 2022; González-Sancho & Picado, 2020; Hernández-Bello et al., 2020; Jha et al., 2023; Londoño & Cañón, 2020) are significant risks for suicidal behavior in adolescents. In addition, out-of-home care, whether in foster or residential care, can have negative effects on the lives of children and adolescents who do not receive adequate care in their family environment. According to Russell et al. (2021), these adolescents have experienced physical, psychological, or emotional harm, or are at risk for such harm, which increases the likelihood of suicide attempts by up to three times compared to those who are not in these situations.

Along the same lines, eight studies (Angelakis et al., 2020; Ati et al., 2020; Cuesta et al., 2021; Howarth et al., 2020; Hernández-Bello et al., 2020; Jha et al., 2023; Quarshie et al., 2020; Russell et al., 2021) have examined adverse life experiences as interpersonal risk factors. Stressful events such as bereavement are associated with increased vulnerability to suicidal ideation and behaviors. In addition, childhood maltreatment, including sexual, physical, and emotional abuse in the family environment, is associated with an increased likelihood of suicidal ideation and planning in adolescents, according to Angelakis et al. (2020), Cuesta et al. (2021), and Hernandez-Bello et al. (2020).

Another relevant aspect of interpersonal risk factors is exposure to suicide (Calderaro et al., 2022; Cuesta et al., 2021; Del Carpio, 2021; Goodday et al., 2019; González-Sancho & Picado, 2020; Hernández-Bello et al., 2020; Jha et al., 2023; Nesi et al., 2021). Loss of parents or primary caregivers to suicide is associated with up to a threefold increased risk of suicide in adolescence (Calderaro et al., 2022; Cuesta et al., 2021; Del Carpio, 2021; Goodday et al., 2019; Hernández-Bello et al., 2020; Jha et al., 2023). Hernández-Bello et al. (2020) also point out that suicidal behavior can have a generational aspect, being transmitted from one generation to the next, regardless of the presence of mental illness. A distinction is made between transmission mechanisms in families where a member has died from causes other than suicide, such as family breakdown or stressful events, and those specific to families with a history of suicide, such as genetic inheritance or imitation (Calderaro et al., 2022).

As mentioned above, adolescents are going through a critical stage in psychological development, where it is essential to have a healthy sense of belonging to peer groups and to expand their social ties. In this context, seven studies examine the influence of the peer group on suicidal behavior in adolescents (Ati et al., 2020; Buelga et al., 2022; Cuesta et al., 2021; Gelvez-Gafaro et al., 2022; González-Sancho & Picado, 2020; Londoño & Cañón, 2020; Wilson et al., 2022). According to Cuesta et al. (2021) and González-Sancho and Picado (2020), the feeling of exclusion from the peer group can have devastating effects on identity development and the formation of future relationships. In addition, four studies (Calati et al., 2019; Cuesta et al., 2021; Domínguez-García & Fernández-Berrocal, 2018; González-Sancho & Picado, 2020) analyze social isolation as a risk factor for suicidal behavior in adolescents. Isolation, lack of interaction with peers, and the absence of trusting social relationships are risk factors associated with adolescent suicide (Domínguez-García & Fernández-Berrocal, 2018; González-Sancho & Picado, 2020). In addition to the quantitative aspect of social isolation, it is important to consider the subjective feeling of loneliness and lack of belonging as more accurate indicators than living alone to analyze their influence on suicidal behavior (Ati et al., 2020; Calati et al., 2019).

##### Community Risk Factors

Community risk factors include circumstances that affect the interaction between the individual and their immediate environment, such as exposure to community violence, local suicide epidemics, or barriers to access to health care. Studying these factors is key when planning and intervening at the public institutional and/or public-community level, mitigating the negative effects they have on adolescents. Only three studies included in this systematic review addressed community factors, and even then, only minimally, which explains their limited presence in the results presented. Although research on the relationship between media, adolescence, and suicide is limited, González-Sancho and Picado (2020) emphasize that sensationalized and biased media coverage of suicides can have significant negative effects. They highlight the Werther Effect as a community risk factor, which can lead to localized contagion or epidemic effects within the population.

Additionally, the impact of the school environment on suicidal behavior in adolescents is evaluated (Castellví et al., 2020; Steare et al., 2023). The schooling stage, which encompasses childhood and adolescence, is marked by significant changes and the transition to adulthood, which can generate anticipated emotional and psychological crises. Studies indicate that if the school does not provide positive support, the risk of crisis and, therefore, the risk of suicide in adolescents increases. Effective support involves fostering greater connection and participation in school, maintaining positive relationships with teachers and peers, and developing competencies to improve academic outcomes (Castellví et al., 2020). In addition, academic pressure has been identified as a relevant factor in forensic studies, finding that suicide rates tend to be lower during non-school periods, such as vacations (Steare et al., 2023).

##### Societal Risk Factors

Societal factors that influence suicidal behavior in adolescents include external events that directly affect their relationship with their environment. Within the SESPM model, these factors include economic recessions, stigma associated with mental health and its treatment, air pollution or infectious diseases, structural poverty, and geographic location. Despite their relevance, the studies included in this systematic review do not address these factors, which explains their absence in the results. It is essential to recognize that, in order to understand suicidal behavior in adolescents, it is necessary to consider not only individual and microsocial factors but also macro-environmental aspects, as these can directly impact the quality of life and well-being of adolescents.

#### Protective Factors

Protective factors against suicidal behavior are resources or conditions, whether biological, environmental, or social, that reduce the probability of suicidal behavior during adolescence (González-Sancho & Picado, 2020). In the social-health field, these factors refer to characteristics present in adolescents or in society that favor the maintenance or recovery of health status, counteract or reduce the effects of risk factors, and/or decrease overall vulnerability to various threats. A common misconception in the scientific literature is that the absence of risk factors alone acts as protection against suicidal behavior. Although reinforcing these protective factors is crucial to reducing risk, these aspects often receive less attention in studies. In this review, only five studies (13.51%) address protective factors (Ati et al., 2020; Hernández-Bello et al., 2020; Benarous et al., 2018; Domínguez-García & Fernández-Berrocal, 2018; Shahram et al., 2020).

##### Individual Protective Factors

Five studies analyze individual protective factors related to suicidal behavior in adolescents (Ati et al., 2020; Benarous et al., 2018; Domínguez-García & Fernández-Berrocal, 2018; Hernández-Bello et al., 2020; Shahram et al., 2020). These factors are defined as individual characteristics, skills, or resources that reduce vulnerability to suicidal behaviors (Shahram et al., 2020). These skills include mental toughness and emotional well-being, as well as resilience through abilities such as coping with adversity, emotional control, adapting to change, enthusiasm for life, planning for the future, and internalizing personal values (Domínguez-García & Fernández-Berrocal, 2018).

As with risk factors, the study of individual protective factors has focused mainly on the psychological domain, considered, from a reductionist perspective, as the area with the greatest individual control. According to Ati et al. (2020) and Hernández-Bello et al. (2020), elements such as finding meaning in life, maintaining adequate nutrition, and participating in positive recreational activities, such as reading or watching movies, are protective factors against suicide in adolescence. In addition, good self-esteem, feeling good about oneself, and employing healthy coping styles are also identified as protective factors (Hernández-Bello et al., 2020). Similarly, emotional control has been examined in four studies: Benarous et al. (2018), Domínguez-García and Fernández-Berrocal (2018), and Shahram et al. (2020). These studies conclude that people with high emotional intelligence tend to form and maintain close relationships, which improves their subjective well-being and the use of effective coping strategies. Expressing emotions and feelings acts as a buffer against suicidal risks and promotes resilience.

Enthusiasm for life, or the will to live, characterized by a sense of engagement and a positive outlook, has been identified by Shahram et al. (2020) as a protective factor against suicide in adolescents. This study suggests that actions such as seeking purpose in life, having reasons to live, and practicing mindfulness or gratitude can moderate risk factors and reduce the likelihood of lifetime suicide attempts. Having reasons to live is considered protective for both adolescent males and females. However, boys tend to have fewer depressive symptoms but also fewer reasons to live, implying a lower risk but with fewer protective factors (Shahram et al., 2020). Only one study (Ati et al., 2020) focuses on studying the influence of personal values, such as faith or religiosity, as a protective factor for suicidal behavior in adolescents. Furthermore, while Hatchel et al. (2019) and Marconi et al. (2023) did not specifically analyze protective factors, they emphasize that a sense of belonging to a peer group and the expansion of social ties are crucial, especially for individuals from minority groups.

A individual protective factor against suicidal behavior in adolescents also involves the adoption of specific behaviors that act as barriers. These behaviors include the ability to seek help, competence in expressing emotions appropriately, use of healthy coping mechanisms, and orientation toward good academic performance. In addition, feeling motivated to establish healthy social relationships, engaging in meaningful activities, managing stress, and adopting a healthy lifestyle are key aspects in protecting against suicidal behavior (Shahram et al., 2020).

##### Interpersonal Protective Factors

Interpersonal protective factors against suicidal behavior in adolescents encompass social conditions and relationships that reduce risk and promote emotional well-being through feelings of connectedness, support, and belonging in the family environment, peer group, and community, as well as cultural and spiritual contexts. In the analysis of these factors, the review includes four studies (12.5%) that examine interpersonal aspects of protection (Ati et al., 2020; González-Sancho & Picado, 2020; Hernández-Bello et al., 2020; Shahram et al., 2020). These studies highlight that a strong social network, family dynamics that foster positive self-esteem, consistent support from parents or caregivers, healthy couple relationships, and religious beliefs and practices provide resources for coping during adolescence and throughout life (González-Sancho & Picado, 2020; Hernández-Bello et al., 2020; Shahram et al., 2020).

##### Societal and Community Protective Factors

Finally, societal and community protective factors include macro-level circumstances such as a stable economy, mental health funding, and institutional policies and resources provided by public authorities. These include crisis hotlines, effective mental health care, and school intervention programs. However, these factors were not examined in any of the studies included in this systematic review.

## Discussion

This research reviewed 37 systematic reviews of the literature, with or without meta-analyses, to identify factors associated with adolescent suicidal behavior and gaps in existing knowledge. According to Cramer and Kapusta (2017), social-ecological theories provide a useful conceptual framework for addressing the complexities of the suicidal phenomenon and its contributing factors. Based on the SESPM, studies were organized into risk and protective factors, classified into individual, interpersonal, community and societal categories. Despite the abundant literature on this topic at a critical stage of development, significant limitations have been identified that hinder a comprehensive understanding of the phenomenon, revealing important gaps in current knowledge.

The recent emergence of critical suicidology has emphasized the importance of considering social, historical, and cultural aspects in the study of suicide (Hagen, 2022; Hjelmeland & Knizek, 2016). This approach challenges traditional paradigms that pathologize suicide by treating it exclusively from an individual perspective (Standley, 2021). Although new approaches suggest that suicide should be understood in broader contexts and not as a static or acontextual phenomenon, the reviewed studies focused mainly on individual or interpersonal factors, excluding events and circumstances external to the individual. This evidence indicates that the current literature continues to conceptualize suicidal behavior from perspectives that tend to pathologize, decontextualize, and individualize suicide, treating it as a purely individual problem rather than a social issue.

Regarding the individual risk factors found, several sociodemographic aspects were highlighted, such as age, gender and belonging to minorities. Traditionally, it has been observed that suicide rates increase with age, but in recent years there has been a significant increase among adolescents aged 15 to 19 years. This increase may be related to psychological, biological, and social changes, such as emotional instability, hopelessness, family adversity, and social alienation (Pelkonen & Marttunen, 2003; Standley, 2021). With respect to gender, the relationship to suicidal behavior in adolescence highlights differential risk factors that require further investigation. Although females have a higher risk of suicide attempts, males have higher rates of completed suicide. This pattern is due, in part, to the fact that males tend to use more lethal methods and are less likely to seek help, which is linked to stereotypes of masculinity that promote independence and emotional strength (Bommersbach et al., 2022; Jones et al., 2023). In addition, belonging to ethnic, gender or sexual orientation minorities has been identified as an important risk factor, since these groups face higher levels of discrimination, stigma and victimization, which increases their vulnerability to depressive disorders and suicide attempts (Kaniuka et al., 2024). In this sense, it is undeniable that the so-called individual factors are deeply influenced by structural, cultural and social conditions, highlighting the need to investigate the specific mechanisms that link these sociodemographic factors with suicidal risk. This relationship is supported by minority stress theory, which suggests that these social minorities experience additional risks that combine with everyday adolescent stressors to increase overall risk (Hatchel et al., 2019; Wang et al., 2023). At the same time, protective factors, such as involvement in supportive communities or mutual support networks, may promote a positive resilience response in these adolescents. Consequently, there is a need to further investigate the specific mechanisms linking sociodemographic factors to suicide risk and to conduct longitudinal studies to better understand the cumulative effects of multiple identities that create unique risk trajectories in adolescents and young adults (Aslan, 2024). This understanding will facilitate the development and implementation of primary prevention strategies in school, family, and professional settings, as well as increased awareness of the complexity of psychological distress experienced by minority youth. In addition, it will be important to incorporate secondary prevention measures to reduce the risk of suicide and to promote collaboration between schools and families to address stigma (Wang et al., 2023).

Consistent with previous research (Weinstein et al., 2021; Xu et al., 2024), most of the reviewed studies identify psychiatric and personal psychological risk factors, such as emotional and mood disorders, risky behaviors, substance use, misuse of social networks, difficulties in emotional control, sleep problems, and unhealthy lifestyle habits, as well as sedentary leisure time and physical or emotional pain. However, despite these associations, the results of the study suggest that these factors alone are not reliable predictors of suicidal behavior and that it is necessary to evaluate them in terms of contextual aspects that exacerbate their negative effects. This limitation is due to the predominance of a biomedical approach that focuses on treating specific symptoms without taking into account the interaction and influence of historical, political, cultural, and social factors. In this sense, and in line with the theory of intersectionality, individual factors should be understood as the product of systemic inequalities resulting from the superimposition of multiple elements (Opara et al., 2020; Robertson et al., 2022). Intersectionality theory adds to the analysis by illustrating how societal and community factors manifest at the individual level, creating disparities in suicide risk. This study argues that although scientific knowledge requires the categorization of problems for analysis, such categorization should not be definitive or static, as all factors are interrelated. Therefore, it is necessary to consider each situation in its context to understand that the causality of suicidal behavior is shaped collectively, resulting from the interaction of broader social, cultural, and historical forces, rather than being solely attributed to individual factors. Following García-Haro et al. (2023), the importance of adopting diverse approaches in research, clinical practice, and policy development to address adolescent suicide from an intersectional perspective is highlighted, overcoming the traditional biomedical paradigm and rigid categorization of factors. Instead, a transdisciplinary approach adapted to the contemporary social and historical context, where multiple factors interact and converge, is proposed.

From the analysis of the results, it is clear that, although risk factors related to the psychological or biological conditions of adolescents have been extensively studied in the last years, attention should also be paid to the risks associated with psychosocial dynamics. The three main spaces of socialization during childhood and adolescence—family, school, and peers—are often the contexts with the highest prevalence of risk (Arango et al., 2024). According to Turecki and Brent (2016), problems in parental relationships and physical abuse, sexual abuse or child neglect emerge as the most significant risk factors. A lack of cohesion, support, security, communication, care, and affection in the family environment is a critical factor for suicidal behavior in adolescents, underscoring the need for assessment and intervention in family functioning and institutional support to mitigate these risks. In the school environment, adolescents without academic or occupational activity and those with academic failure present a markedly increased risk of suicide attempts, which is exacerbated by excessive parental pressure on academic performance (Rai, 2021). While social integration and peer groups may act as protective factors, social isolation, peer pressure, and loneliness—characterized by a lack of interaction and trusting relationships—are prominent risk factors for suicide in adolescents (Van Orden et al., 2010). In this sense, the school context constitutes a fundamental area for planning actions and providing training resources for teachers. According to Singer et al. (2019), the importance lies in identifying these maladjustments and addressing them through action protocols and coordination with welfare areas, particularly with social services, education, and health systems, which are better prepared to address serious difficulties affecting the biopsychosocial development of adolescents.

The results of the review also indicate the need to redirect research efforts toward elements that protect adolescents from suicide. Most of the literature reviewed has focused on deficit-based models, which primarily identify risks associated with adolescent suicide. This limited perspective restricts the development and effectiveness of interventions by not adequately addressing the resources and conditions that can prevent suicide. According to García-Mollá et al. (2024), it is paramount to identify and value protective factors, as they not only help reduce the probability of suicidal behaviors, but also strengthen the dynamics that lead to success, improve coping strategies, facilitate the establishment of evaluative intervention goals, and allow the coordination of actions with different welfare systems. Positive psychology and strengths-based models offer a more effective approach by reinforcing individual and collective potential (Fonseca-Pedrero et al., 2022; O’Keefe et al., 2022). Therefore, it is critical to shift the focus to the identification and promotion of protective factors to develop more effective community prevention strategies. In this regard, it is essential to train mental health professionals in integrative intervention approaches that not only take risks into account but also turn them into strengths, enhancing the potential of adolescents.

This review reveals an alarming lack of studies analyzing societal and community factors related to adolescent suicidal behavior directly, which has important implications for research, policy, and practice. The lack of analysis on aspects such as economic downturns, stigma associated with mental health and suicide, structural poverty, community violence, and barriers to access to health care impedes a comprehensive understanding of the suicidal phenomenon and limits the development of new theories and models. This omission impairs the formulation of effective public policies and limits the design of interventions adapted to local and community realities, affecting equality in access to resources and multi-sectoral coordination. Collaboration between educational institutions, health, and community organizations is essential to implement a comprehensive approach to reduce suicide rates, improve adolescent well-being and understand the unique risk experiences of adolescents. The lack of literature on societal and community factors highlights the need to investigate challenges in systems of care for adolescent suicidal behavior. Identifying gaps and opportunities in institutions that intervene with adolescents is essential to guide future research and develop evidence-based solutions, maximizing the impact of public policies for care.

### Limitations of the Study

This review has some limitations. First, the keywords and databases selected may have omitted relevant information, which limits the completeness of the search. Additionally, publication bias is another potential constraint. This bias implies that evidence published in peer-reviewed journals may be biased toward studies with positive results or toward predominant areas in the literature, such as biomedical sciences or psychology, which may not fully reflect the entire body of available research.

## Conclusions

This review of 37 studies of adolescent suicidal behavior reveals a troubling lack of analysis of societal and community risk and protective factors. Although the SESPM emphasizes the importance of these factors, the current literature focuses primarily on individual and interpersonal deficits, which limits a comprehensive understanding of the problem and hinders the effectiveness of interventions. Future research needs to integrate elements related to the physical and social environments that influence mental health and behavior, as well as enhance adolescents’ resources and strengths. This integration will support the development of more comprehensive public policies and community strategies that address the real needs and potential of adolescents, ultimately improving the effectiveness of interventions and their overall well-being.

Finally, this study proposes the need to move toward a transdisciplinary and relational model that views adolescent suicide as a collective phenomenon influenced by the dialectic between agency and structure, between individuality and social context, and between biology and culture. Only through an approach that integrates these dimensions and recognizes the complex interaction between individual and systemic factors will it be possible to develop more effective and equitable interventions that are tailored to local contexts and the specific needs of adolescents at risk.
